# Chronische posttraumatische Schulterinstabilität

**DOI:** 10.1007/s00113-022-01283-9

**Published:** 2023-02-02

**Authors:** S. Bauer, B. Dietz, P. Collin, L. Neyton, W. Blakeney, M. Zumstein

**Affiliations:** 1 Hôpital de Morges, Ensemble Hospitalier de la Côte, Chemin du Crêt 2, 1110 Morges, Schweiz; 2 St. Josefs-Hospital, Wiesbaden, Deutschland; 3 Clinique Victor Hugo, Paris, Frankreich; 4 Centre Orthopédique Santy, Lyon, Frankreich; 5 Royal Perth Hospital, Perth, Australien; 6 Inselspital und Orthopädie Sonnenhof, Bern, Schweiz; 7 School of Surgery, University of Western Australia, Perth, Australia

**Keywords:** Schulterluxation, Bankartstabilisierung, Latarjet-Operation, Arthropathie, Rezidiv, Shoulder dislocation, Bankartstabilisierung, Latarjet operation, Arthropathy, Recurrence

## Abstract

Die chronische posttraumatische Schulterinstabilität ist durch traumaassoziierte, rezidivierende Luxationen charakterisiert. Es wird kontrovers diskutiert, wie Risikofaktoren zur Auswahl zwischen arthroskopischer Bankart-Reparatur mit Kapselshift (ABRK), Latarjet- und Alternativtechniken gewichtet werden sollten. Als Risikofaktoren gelten Lebensalter, Hyperlaxizität, Sportprofil und Knochenverlust. Die ABRK geht mit hoher Patientenzufriedenheit und Rückkehr zum Sport einher. Rezidive treten, assoziiert mit Risikofaktoren, noch nach mehreren Jahren auf. Latarjet- oder Knocheneingriffe erzielen eine hohe Patientenzufriedenheit und dauerhafte Stabilität im Revisionsfall, können aber auch als Ersteingriff bei entsprechendem Risikoprofil indiziert sein, wobei von einer höheren Rate meist leichter Komplikationen berichtet wurde. Jegliche Techniken unterliegen einer ernstzunehmenden Lernkurve. Bei korrekter Durchführung scheint das Risiko einer operationsbedingten Arthroseentwicklung nicht erhöht zu sein; diese wird vielmehr durch die Anzahl der Rezidivluxationen und traumatische Gewalteinwirkung beeinflusst.

## Abstract (en)

Chronic posttraumatic shoulder instability is characterized by trauma-associated, recurrent dislocations. Surgical treatment is indicated in most cases but it remains controversial how risk factors should be weighted to decide between arthroscopic Bankart repair (ABR), Latarjet or alternative procedures. Known risk factors for recurrence are patient age, hyperlaxity, sports profile and bone loss. Surgical techniques are discussed in detail. The ABR leads to high patient satisfaction and return to sports; however, in association with risk factors, recurrent dislocations are seen even several years later. Latarjet or bone block procedures lead to high patient satisfaction, sustainable stability as a revision procedure, but can also be primarily indicated for chronic instability depending on risk factors. Early complications are more frequent but of a minor nature in most cases. All techniques are known for a serious learning curve. If performed well, they do not seem to increase the risk of arthritis, which is most affected by the number of lifetime dislocations and higher energy trauma.

## Lernziele

Nach Lektüre dieses Beitrages kennen Sie die

- Vor- und Nachteile der verschiedenen Operationsverfahren,
- Rezidivrisikofaktoren und ihre Bedeutung,
- das Ausmaß der Patientenzufriedenheit und die Langzeitergebnisse,
- Resultate bei Sportlern,
- Faktoren, die zur Omarthrose führen können.

## Einleitung

Die chronische posttraumatische Schulterinstabilität kann nach einer Luxation mit erforderlicher Fremdreposition und entweder traumatischer Knochen- (humeral oder glenoidseitig) oder kapsuloligamentärer Läsion diagnostiziert werden, sofern weitere Luxationen, symptomatische Subluxationen oder eine dauerhaft störende Luxationsangst („apprehension“) vorliegen. Klinische Befunderhebung, bildgebende Untersuchungen und Klassifikationen wurden in der hervorragenden Arbeit von Lichtenberg dargestellt [1].

### Fallbeispiele

**Figure 1:**
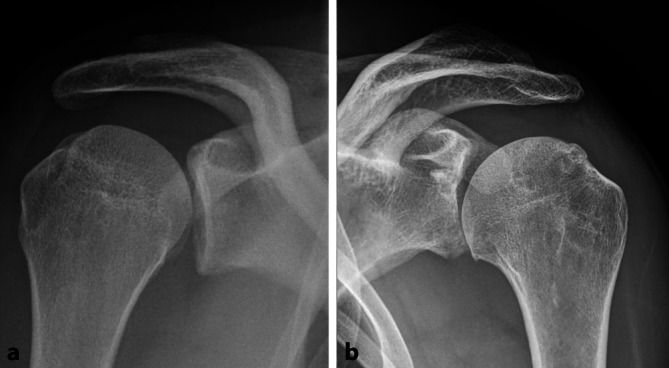

a): Ein 20-jähriger, höherklassiger Handballer (Kreisläufer), 2. Vorstellung. Vor 12 Wochen rechtsseitige Erstluxation bei Gegnerblockade der Wurfbewegung. Notfallreposition. Nun Zweitluxation. Untersuchung: Außendrehung 90°, linksseitige Hyperabduktion. Anterior-posteriore Röntgenaufnahme: Abb. 1a.
b): Ein 32-jähriger Fußballer (Verbandsliga), traumatische linksseitige Erstluxation mit 17 Jahren, nun Notfallreposition, zuvor 8 Luxationen und 3 Repositionen durch Helfer. Keine Hyperlaxizität. Anterior-posteriore Röntgenaufnahme: Abb. 1b.
1.: Was erkennen Sie auf den Röntgenbildern?
2.: Wie erheben Sie den Instability Severity Index Score (ISIS)?
3.: Welche Operationsmethoden empfehlen sich?
4.: Wie ist die Prognose bezüglich der Beweglichkeit und Entwicklung einer Arthrose einzuschätzen?

## Chirurgische Behandlungsoptionen

### Arthroskopische Bankart-Reparatur mit Kapsel-Shift (ABRK)

Die arthroskopische Bankart-Reparatur mit Kapsel-Shift (ABRK) wird im überwiegenden Teil der Fälle mit 3 Ankern durchgeführt und kann in Seitenlage mit Doppeltraktion oder sitzender Position erfolgen. Der **kapsuloligamentäre Komplex**

kapsuloligamentäre Komplex

wird vom M. subscapularis gelöst und mobilisiert. Die labrale Insertion wird angefrischt, und es wird darauf geachtet, dass die Anker am Glenoidrand nicht medialisiert werden und somit eine Labrumwulstbildung („bumper“) in knotenloser oder Knotentechnik ermöglichen. Zuvor wird die meist ausgedehnte Kapselläsion durch Verschiebung („Shift“) mit versetzter Stichtechnik wieder auf eine normale Länge gebracht [2]. Nach einer ABRK wurden eine hohe Patientenzufriedenheit und Rückkehr zum Sport dokumentiert [3]. Es zeigen sich jedoch Rezidivluxationen, assoziiert mit Risikofaktoren [4, 5].

#### Merke

ABRK: hohePatientenzufriedenheit und Sportrückkehr, jedoch Rezidivluxationen bei Risikoprofil.

### Arthroskopische Bankart-Reparatur mit Kapsel-Shift + Remplissage

Als Lösung für das Problem einer sich **verhakenden Hill-Sachs-Läsion**

verhakenden Hill-Sachs-Läsion

(HSL) entwickelte Wolf die Hill-Sachs-Remplissage [6]. Die posterosuperiore Kapsel und Infraspinatussehne werden mithilfe eines Ankers in die HSL genäht, die dadurch extraartikulär verbleibt und sich nicht mehr einhakt. Ein systematischer Review verglich die arthroskopische Bankart-Reparatur mit Kapsel-Shift + Remplissage (ABRK + R) mit Latarjet-Eingriffen [7]. In Studien mit glenoidseitigem Knochenverlust (KV) von 10–15 % zeigte sich eine signifikant höhere Instabilitätsrate für ABRK + R (6,1–13,2 % vs. 0–8,2 %). Eine Metaanalyse ermittelte eine signifikant niedrigere Rate von Instabilitätsrezidiven für die ABRK + R im Vergleich zum isolierten ABRK [8]. Dies wurde in einer randomisierten kontrollierten Studie von Mac Donald bestätigt (18 % vs. 4 %, [9]). Die Technik sollte bei Wurfsportlern vermieden werden, da von einer **eingeschränkten Außenrotation**

eingeschränkten Außenrotation

auszugehen ist [10].

#### Merke

- Eine Remplissage ist beim Wurfsportler zu vermeiden.
- Anwendung nur bei subkritischem KV (< 10 %).

### Freie Knochenblockverfahren nach Resch und Scheibel

Aufgrund des erhöhten Luxationsrisikos bei KV am Glenoid entwickelte Resch die **J‑Span-Technik**

J‑Span-Technik

als chirurgische Alternative [11, 12, 13]. Ein unikortikaler J‑Span wird aus dem Beckenkamm entnommen. Nach Tenotomie der Subskapularissehne und Arthrotomie wird mit einem Meißel eine Spalte in die Skapula eingeschlagen, in die der J‑Span impaktiert wird. Danach erfolgt die **Subskapularisrefixierung**

Subskapularisrefixierung.

Die Langzeitnachuntersuchung von 35 Schultern (min. 15 Jahre) zeigte eine gute Reluxationsrate (*n* = 1; 2,9 %) bei verbleibender Apprehension in 23 % der Fälle [14].

Scheibel et al. veröffentlichten die arthroskopische Rekonstruktion von glenoidseitigem KV mithilfe eines **autologen Knochenblocks**

autologen Knochenblocks,

bioabsorbierbarer Schraubenfixierung und Bankart-Reparation [15]. Die Nachuntersuchung nach mindestens 5 Jahren ergab in 2 Fällen (14 %) eine operationsbedürftige Rezidivinstabilität bei einer erneuten Luxation (7 %). Der **subjektive Schulterwert**

subjektive Schulterwert

(Subjective Shoulder Value, SSV) betrug im Mittel 87 % [16]. Computertomographische Untersuchungen zeigten ein **Remodeling**

Remodeling

des Glenoids zur anatomischen Birnenform („pear shape“). Nach Einbringen eines Allografts stellte sich in 14 % der Fälle ein erneuter KV durch Resorption mit Apprehension in 30 % der Fälle (*n* = 3) bei einer **Rezidivsubluxation**

Rezidivsubluxation

ein. Die Glenoidform konnte nicht wiederhergestellt werden.

#### Merke

Freie Knochenaugmentationen: Autologer Knochen erzielt bessere Ergebnisse als allogener Knochenaufbau.

### Latarjet-Patte-Operation nach Walch

Nach 40-jähriger klinischer Erfahrung mit über 3500 Korakoid-Transfer-Eingriffen und Weiterentwicklung zur Walch-Technik kann das Verfahren als Standard der Latarjet(LAT)-Techniken angesehen werden. Die Langzeitergebnisse sind sehr gut (1 % Reluxationen) und konnten von Gerber in gleicher Technik reproduziert werden [17]. In unserem Technikbeitrag (S.Bauer und Mitarbeiter) „Latarjet-Patte nach Walch“ in der vorliegenden Ausgabe wird die Technik detailliert beschrieben. Hauptvorteil der Methode ist die Stabilisierung der Schulter durch den **Schlingeneffekt**

Schlingeneffekt

und zusätzlichen **Hängematteneffekt**

Hängematteneffekt

(Conjoint-Sehne und unterer Subskapularis, s. Technikbeitrag), die bei freier Knochenaugmentation nicht zum Tragen kommen. Die Lernkurve dieser offenen Technik sollte nicht unterschätzt werden. Auch bedingt durch variable LAT-Verfahren wird von Komplikationsraten bis zu 30 % berichtet, bei denen es sich jedoch größtenteils um leichte Vorfälle handelt [18]. Beim Erlernen der Walch-Technik wird auf die Standardisierung Wert gelegt; Gefahren und Komplikationsrisiken sollten bekannt sein [19].

#### Merke

Latarjet-Patte-Operation nach Walch: standardisierter Korakoidtransfer mit niedriger Langzeitrezidivrate (1–5 %).

## Risikofaktoren für Rezidive nach Weichteilstabilisierungen

### Nachuntersuchungszeit („length of follow-up“)

**Figure 2:**
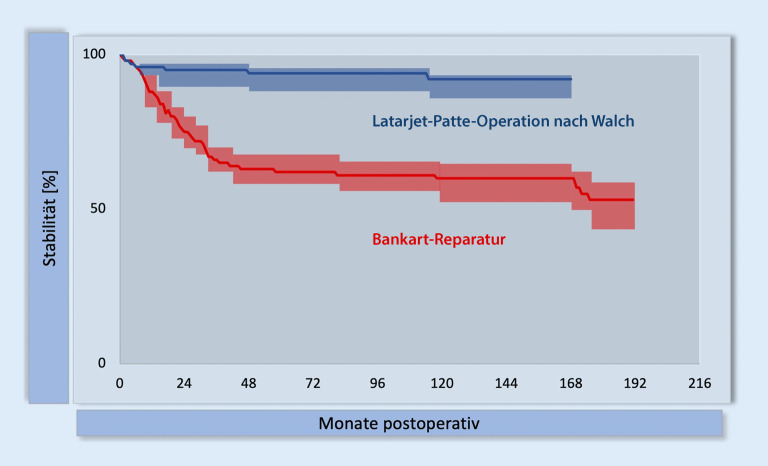

Die Dauer der Nachuntersuchung stellt eine bedeutende Risikovariable für Rezidive dar. Dies wurde in 3 Vergleichsstudien (Abb. 2 und 3) gezeigt [17, 20, 21]. Bei alleinigen kapsulolabralen Stabilisierungen lassen sich über Jahre hinweg Rezidive beobachten. Eine randomisierte kontrollierte Studie (RCT) mit jungen Männern (Alter < 25 Jahre) und chronischer Instabilität zeigte bereits nach 2 Jahren eine signifikant niedrigere Luxationsrate nach LAT-Eingriffen [21].

**Figure 3:**
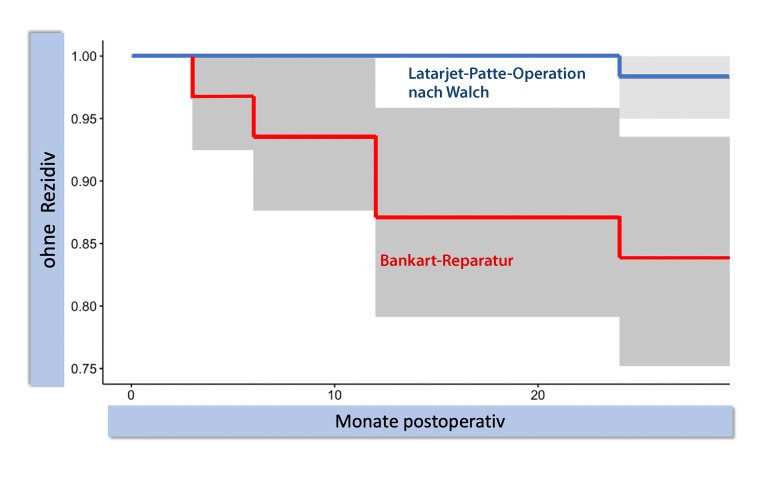

#### Merke

Bei alleiniger kapsulolabraler Stabilisierung treten Rezidive auch langfristig im Verlauf noch auf.

### Lebensalter der Patienten

Bei jüngeren Patienten wurde eine erhöhte Rezidivrate nach einer ABRK dokumentiert [22]. Balg und Boileau nahmen eine Alterstrennung (< 21 Jahre) vor, die signifikante Unterschiede bezüglich einer Rezidivinstabilität ergab (*p* = 0,001, [23]). Das erhöhte Rezidivrisiko nach einer ABRK bei Patienten unter 21 Jahren wurde in mehreren Langzeitstudien nachgewiesen (Rezidivraten 54 % und 39,1 %, [3, 4]). Der bereits angeführte RCT junger Männer bestätigt diese Nachuntersuchungen (Rezidive: 22 % nach einer ABRK im Vergleich zu 2 % nach einer LAT-Operation, [21]).

#### Merke

Erhöhtes Rezidivrisiko nach einer ABRK bei jungen Patienten.

### Knochenverlust an Glenoid und Humerus

Ein KV kann auf dem **anterior-posterioren Röntgenbild**

anterior-posterioren Röntgenbild

erkannt, jedoch schlecht quantifiziert werden (Abb. 1a, b) und wurde als Hauptkriterium in den **Instability Severity Index Score**

**Figure 4:**
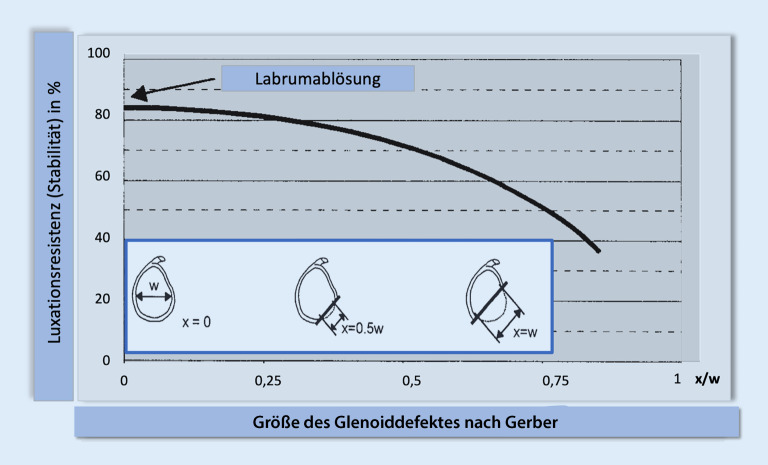

Instability Severity Index Score

(ISIS) mit maximal 4 möglichen Punkten (40 % des maximalen Gesamt-Scores [GS]) integriert (Abb. 4; [23]). Zur quantitativen Bestimmung des KV ist eine **Computertomographie**

Computertomographie

notwendig [24]. Die Form des Glenoids, und ob ein KV vorliegt, kann bereits mithilfe der sagittalen und axialen **Magnetresonanztomographie**

Magnetresonanztomographie

abgeschätzt werden.

Bereits 1998 klassifizierte Bigliani **Glenoidrandläsionen**

Glenoidrandläsionen

und empfahl, KV von mehr als 25 % mithilfe einer LAT-Operation zu versorgen [12]. Der kritische Grenzwert (KGW) wurde in den letzten Jahren sukzessive zwischen 13,5 und 17,3 % nach unten korrigiert und kann gerundet zwischen 10-20 % angesiedelt werden [25, 26].

Im Gegensatz zu dem Versuch, einen KGW zu definieren, demonstrierte Gerber ein gegenläufiges Verhältnis zwischen Gelenkstabilität und KV (Abb. 3), wobei ein KV jeglichen Ausmaßes nicht vollständig von einer kapsulolabralen Stabilisierung kompensiert werden kann. Bei bereits geringem KV verbleibt eine alleinig kapsulolabral reparierte Schulter mit einer Stabilität von weniger als 100 %, mit einem Stabilitätsverlust je nach Ausmaß des KV [13].

#### Merke

- Nach Gerber führt bereits jeglicher geringer KV zur Verringerung der Luxationsresistenz.
- Der kritische KV wird heute bei 10 –20 % angesetzt.

Yamamoto und Itoi untersuchten die Bedeutung von HSL und führten das **„Off-track“-Konzept**

„Off-track“-Konzept

des Glenoids ein [27]. Die Kontaktzone des Glenoids und des Humeruskopfes bei Armbewegungen wird als **„glenoid track“**

**Figure 5:**
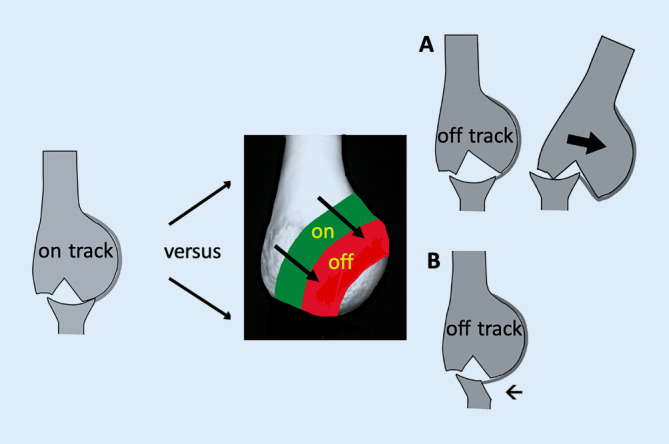

„glenoid track“

bezeichnet. Eine „Off-track“-HSL verlässt die Glenoidfläche bei einer Armbewegung mit dem Risiko der Luxation und Verhakung (Abb. 5*A*, *B*). Die Prävalenz einer „Off-track“-HSL beträgt ca. 7 % [28]. Die „Off-track“-HSL kann mithilfe einer LAT-Operation oder einer Remplissage behandelt werden, wobei eine Remplissage bei Werfern, z. B. Handballern, mit dem Bedarf einer erhöhten Außenrotation des Wurfarmes aufgrund möglicher Außenrotationseinschränkung nicht angewandt werden sollte [10].

#### Merke

Eine „Off-track“-HSL liegt bei ca. 7 % der Patienten mit chronischer traumatischer Instabilität vor.

### Sportprofil

Ein erhöhtes Auftreten der traumatischen Schulterinstabilität wurde für **Zweikampfsport**

Zweikampfsport

wie Rugby, American Football, Australian Football, Handball, Basketball, Eishockey und Fußball ebenso wie für **Kampfsportarten**

Kampfsportarten

und Ringen dokumentiert. Dies gilt ebenso für Sportarten mit **Sturzgefahr**

Sturzgefahr

wie Skateboarden, Snowboarden, Surfen, Skifahren, Überkopf- und Wurfsportarten [29].

Wettkampfsport im Vergleich zu Breitensport wurde im ISIS mit 2 Punkten bewertet (20 % GS) und war als Variable statistisch signifikant (*p* = 0,031, [23]).

#### Merke

Wettkampfrisikosport ≙ Hauptkriterium für Rezidive nach alleiniger ABRK.

### Hyperlaxizität

Hyperlaxizität wurde bei beidseitiger Schulterinstabilität beobachtet [30, 31]. Im ISIS wird sie mithilfe des **Walch-Coudane-Tests**

Walch-Coudane-Tests

(Außenrotation mit angelegtem Arm > 85°) und des **Gagey-Tests**

Gagey-Tests

(Unterschied der Hyperabduktion bei fixierter Scapula > 20° im Seitenvergleich) bewertet (ein Punkt, 10 % GS) [23].

#### Merke

Außenrotation und Hyperabduktion dienen der Erfassung einer spezifischen Schulterhyperlaxizität.

### Kritische kapsuloligamentäre Verletzungen

Die anteriore labroligamentäre „sleeve avulsion“ (ALPSA) ist mit einem erhöhten Rezidivrisiko nach alleiniger kapsulolabraler Reparation vergesellschaftet [32]. Dies scheint mit einer erhöhten **anterioren Kapselbanddehnung**

anterioren Kapselbanddehnung

assoziiert zu sein. Für arthroskopische Eingriffe bei humeraler Avulsion der glenohumeralen Ligamente (HAGL) wurden bei Spitzensportlern trotz allgemein guter Resultate Folgezustände mit Nichterreichen des vorherigen Sportniveaus dokumentiert [33]. Humerale Avulsionen der glenohumeralen Ligamente können mit der LAT-Technik effektiv behandelt werden [34]. Dies gilt auch für ALPSA-Läsionen.

#### Merke

Die ALPSA- und HAGL-Läsion stellen kritische traumatische Weichteilverletzungen dar.

### Schweregradscore einer Instabilität nach Boileau

**Figure 6:**
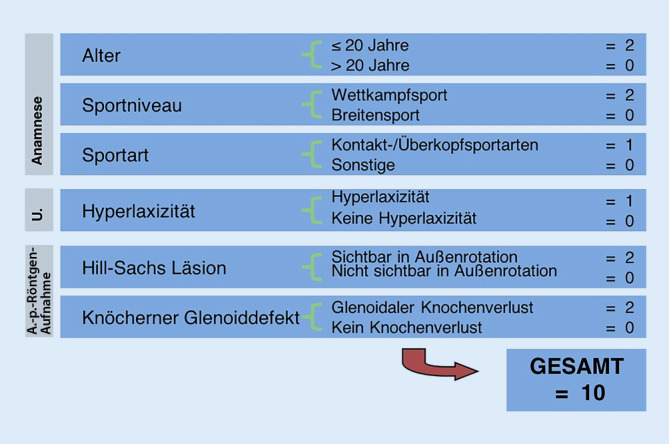

Der ISIS beinhaltet 6 Kriterien (Abb. 6), wobei 3 Hauptkriterien 60–80 % (6 bis 8/10 Punkte) des möglichen GS ausmachen: Alter (2 Punkte), Wettkampfsport (2 Punkte) und KV (Hill-Sachs-Läsion + Glenoid: 2 bis 4 Punkte) [23]. Bei der Erstpublikation 2007 wurde eine LAT-Operation bei einem ISIS von mehr als 6 Punkten empfohlen. Aufgrund einer erhöhten Rezidivrate wurde dieser Cut-off-Wert 2014 auf 3 Punkte korrigiert [20]. Bei Vorliegen von kritischem KV am Glenoid oder 2 Hauptkriterien (4 Punkte) oder einem Hauptkriterium und 2 Nebenkriterien (4 Punkte) ist eine LAT-Operation zu empfehlen.

#### Merke

Als Hauptkriterien für ein erhöhtes Rezidivrisiko gelten Patientenalter, Wettkampfsport und Knochenverlust.

## Patientenzufriedenheit

Die ABRK wurde bezüglich der Patientenzufriedenheit mit der Latarjet-Technik nach Walch verglichen (minimales Follow-up von 6 Jahren, [17]). Die Unterkohorten erfolgreicher ABRK- und Latarjet-Eingriffe nach Walch, ohne jegliche postoperative Instabilität in beiden Gruppen, gaben eine hohe Zufriedenheit der Patienten nach beiden Operationsverfahren an. Die Patienten nach einer Latarjet-Walch-Operationen waren zwar signifikant zufriedener als nach einer ABRK (durchschnittlicher SSV 91 % vs. 87 %; *p* = 0,002), es ist jedoch nicht sicher, ob dieser Unterschied klinische Relevanz besitzt. Die Ergebnisse nach einer ABRK stehen im Einklang mit jenen der arthroskopischen, beckenkammaugmentierten ABRK (durchschnittlicher SSV: 87 %, [16]).

### Merke

Patienten zeigen eine hohe subjektive Zufriedenheit nach Bankart‑, Latarjet- und Alternativverfahren.

## Sportrehabilitationsparameter

Es gibt 4 wichtige Kriterien des „Return to Sports“ (RTS): 1. prozentualer Anteil der Sportrückkehrer (RTS-Rate), 2. Zeit bis zum Wiedereinstieg (RTS-Zeit) 3. Rate desselben Niveaus (RTS-Niveaurate), 4. Stabilität auf Zeit.

Der Vergleich von 120 Sportlern, die mit dem ABRK- oder Latarjet(LAT)-Verfahren operiert wurden, lieferte signifikante Unterschiede [29]. Die **Return-to-Sports-Rate**

Return-to-Sports-Rate

(Training) nach der LAT-Operation war signifikant höher, und die **Return-to-Sports-Zeit**

Return-to-Sports-Zeit

war signifikant kürzer (*p* = 0,031 und 0,034). Die RTS-Zeit (Training) betrug nach LAT-Operationen 5,1 Monate und nach einer ABRK 6,4 Monate. Die **Rezidivrate**

Rezidivrate

nach LAT-Verfahren betrug 2,5 % und nach ABRK 17,9 % (*p* < 0,001).

Bei Rugbyspielern erzielten ABRK und LAT-Operationen eine RTS-Rate von 92 % für beide Gruppen bei signifikant besserer Stabilität nach LAT-Operationen (Rezidivrate 4 % vs. 20 %, *p* = 0,01, [35]). Eine niedrige Rezidivrate (0 %) und eine kurze RTS-Zeit von 3 bis 4 Monaten wurden in einer Langzeitkohortenstudie für Rugbyspieler bestätigt [36].

Bezüglich des **Return-to-Sports-Niveaus**

Return-to-Sports-Niveaus

ermittelten LAT-Operation-Einzelstudien für Fußballer eine RTS-Zeit von 3 bis 7 Monaten, eine RTS-Rate von 96–100 %, eine RTS-Niveaurate von 71–100 % und eine Rezidivrate von 2,6 % [37, 38].

Eine Vergleichsstudie (ABRK vs. LAT-Operation) im Australian Football erhob für Profis eine RTS-Rate über 90 % für beide Gruppen bei einer Rezidivrate von 0 % für LAT und von 19 % für ABRK [39].

### Merke

- Return-to-Sports-Raten und RTS-Niveaurate sind nach ABRK und LAT-Operationen gut.
- Die RTS-Zeit und Langzeitrezidivrate sind nach LAT-Operationen besser.

## Revisionschirurgie nach postoperativer Rezidivinstabilität

Für LAT-Erstoperationen wurden bessere Ergebnisse im Vergleich zum Latarjet-Zweiteingriff nach kapsulolabraler Stabilisierung veröffentlicht [40]. In einer Studie aus dem Jahr 2021 waren die RTS-Rate und die subjektive Zufriedenheit nach LAT-Operationen bei Patienten mit großer HSL vermindert, wobei 58 % dieser Patienten zuvor mit einer arthroskopischen, kapsulolabralen Stabilisierung behandelt wurden sowie 70 % der Patienten mehr als 4 und 51,7 % der Patienten mehr als 9 Luxationen erlitten hatten. Patienten mit höherer Luxationszahl hatten größere HSL [41].

Eine systematische Übersicht über die Revisionschirurgie nach fehlgeschlagener Erstoperation unter Einschluss von 1110 Schultern zeigte eine **Folgeinstabilität**

Folgeinstabilität

von 3,8 % nach einer LAT-Operation, 16 % nach einer ABRK und 20,8 % nach einer Knochenaugmentation vom Beckenkamm [42]. In Anbetracht dieser Daten empfiehlt sich ein Latarjet-Eingriff zur Vermeidung von Rezidivluxationen mit progressivem KV im Revisionsfall. Bei chronischer traumatischer Instabilität scheinen HSL ab einer gewissen Größe nicht mehr kompensierbar zu sein [43].

### Merke

Hohe Luxationszahl, große HSL: schlechtere LAT-Operation-Ergebnisse.

## Instabilitätsarthrose

Eine bedeutende Arbeit von Hovelius et al. [44] wies folgende Faktoren für eine Instabilitätsarthrose nach:

1. rezidivierende Instabilität,
2. höheres Alter bei Erstluxation,
3. Instabilität bei Alkoholismus,
4. Sportarten mit Gewalteinwirkung.

Hovelius et al. wiesen auch darauf hin, dass ein laterales Überstehen des Knochenblocks nach Bristow-Latarjet-Operationen mit erhöhtem **Arthroserisiko**

Arthroserisiko

assoziiert zu sein scheint [45]. Dies wurde in Walchs Langzeitserie bestätigt [46]. Im Langzeitverlauf besteht kein Vorteil für ABRK-Eingriffe (Arthroseprävalenz von 36,8 %) im Vergleich zum LAT-Eingriff nach Walch (23,5 %). In Langzeitnachuntersuchungen der LAT-Technik nach Walch fand sich eine geringe Arthroseprogredienz, sofern kein laterales Überstehen des Knochenblocks vorlag [43, 46]. Reider zieht die Schlussfolgerung, dass eine korrekt ausgeführte LAT-Operation das Arthroserisiko nicht zu erhöhen scheint [47].

### Merke

Korrekt ausgeführte ABRK und LAT-Operation: kein kausaler Zusammenhang mit einer Arthroseentwicklung.

### Auflösung der Fallbeispiele

1. Beim Handballer in Abb. 1a sind eine HSL und eine leichte Verwaschung der inferioren Glenoidkortikalis zu erkennen. Es handelt sich um eine Aufnahme in leichter Innenrotation entgegen der Erhebung nach Boileau. Beim Fußballer in Abb. 1b ist eine HSL in Neutralstellung, der Verlust der inferioren Glenoidkortikalis (KV am Glenoid) und eine leichte Instabilitätsarthropathie zu erkennen.
2. Handballer: 6 bis 7 Punkte je nach KV am Glenoid; Fußballer: 4 bis 6 Punkte je nach HSL.
3. Bei beiden Sportlern LAT-Operation nach Walch oder ABRK mit autologem Knochenaufbau.
4. Bei dauerhafter Stabilisierung der Schultern mit korrekter Technik ist das Arthroserisiko beim Handballer nicht erhöht, beim Fußballer kann von einer Limitierung der Arthroseprogression ausgegangen werden.

## Fazit für die Praxis

- Eine alleinige Weichteilstabilisierung sollte bei glenoidalem Knochenverlust mit Vorsicht angewandt werden.
- Bei der operativen Indikationsstellung zur Behandlung der chronischen posttraumatischen Instabilität sind die genaue Evaluierung des Knochenverlustes an Glenoid und Humerus, die Berücksichtigung des Patientenalters und die Einschätzung des Risikoprofils im Wettkampfsport sinnvoll.
- Rezidivierende Luxationen höherer Zahl sind mit einer Arthroseprogredienz assoziiert und sollten durch eine adäquate Auswahl des Operationsverfahrens und die Beratung des Patienten vermieden werden.

## CME-Fragebogen

In ihrer Sprechstunde stellt sich ein 27-jähriger Patient mit traumatischer Schulterinstabilität und 3 vorausgegangenen Luxationen vor. Er betreibt lediglich Breitensport. Überkopfsportarten werden verneint. Es zeigt sich eine kleine „On-track“-Hill-Sachs-Läsion mit minimalem glenoidalen Knochenverlust (< 5 %). Bei der klinischen Untersuchung ergibt der Gagey-Test einen negativen Befund. Welche Therapiemethode könnten Sie diesem Patienten empfehlen?

Eine Latarjet-Operation, da der Instability Severity Index Score (ISIS) > 3 beträgt.

Eine konservative Therapie, da die Reluxationswahrscheinlichkeit unter 10 % beträgt.

Eine arthroskopische Bankart-Operation ist eine gute Wahl mit niedrigem Komplikationsrisiko.

Eine ossär autolog augmentierte Bankart-Operation bei relevantem Knochenverlust.

Eine Bankart-Operation mit Remplissage.

Wie lautet Gerbers Postulat zur Beurteilung des glenoidalen Knochenverlusts (KV) bei chronischer Instabilität, basierend auf biomechanischen Untersuchungen?

Der kritische KV beträgt 25 %.

Der KV am Glenoid kann mithilfe einer Remplissage + Bankart-Operation kompensiert werden.

Bereits ein geringer glenoidaler KV reduziert die Luxationsresistenz nach einer Bankart-Operation.

Zur quantitativen Bestimmung des KV ist ein konventionelles Röntgenbild notwendig.

Glenoidaler und humeraler KV sollten in der Addition unter 30 % gehalten werden.

Welche Faktoren gehen mit dem höchsten Risiko einer Rezidivluxation nach einer Bankart-Operation einher?

Knochenverlust, Alter > 50 Jahre, Turnsport

Knochenverlust, Alter > 30 Jahre, Kontaktsport

Knochenverlust, Alter < 21 Jahre, Kampfsport

Knochenverlust, Alter < 30 Jahre, Überkopfsport

Knochenverlust, Alter < 21 Jahre, Tanzsport

Was ist der Hauptvorteil und die Hauptkomponente der Stabilisierung bei der Latarjet-Operation?

Die Schraubenfixierung ist mechanisch stabiler als die Ankerfixierung.

Der Knochentransfer ist mechanisch stabiler als chronisch geschädigtes Gewebe.

Die Vergrößerung der Glenoidfläche durch den Knochen ist entscheidend.

Der Schlingen- (Conjoint-Sehne) und Hängematteneffekt (unterer Subskapularis).

Die zusätzliche Stabilisierung der Kapsel durch die Naht ans korakoakromiale Band.

Wann beobachtet man subjektive schlechtere Ergebnisse nach einer Latarjet-Operation?

Bei jungen Patienten oder Frauen

Bei Rugby- und Australian-Football-Spielern

Bei Werfern und Gewichthebern

Bei großer Hill-Sachs-Läsion und hoher Luxationszahl

Bei Adipositas

Wie wird die Subskapularissehne (SSS) beim J‑Knochenblock-Transfer nach Resch behandelt?

Man kann beim offenen Verfahren auf eine Tenotomie der SSS oder Ablösung verzichten.

Der J‑Span nach Resch in offener Technik beinhaltet die komplette Tenotomie der SSS.

Ein Split der SSS ohne Tenotomie wird oftmals angewandt.

Die SSS wird mithilfe eines Schraubankers am J‑Span refixiert.

Ein Vorteil des J‑Spans ist, dass die SSS nicht im Zugangsgebiet liegt.

Welches sind die häufigsten Risikofaktoren für die Entstehung einer Omarthrose bei Schulterinstabilität?

Betroffenheit der dominanten Schulter, fortgeschrittenes Lebensalter bei Erstluxation, Nikotinkonsum, Sportarten mit Gewalteinwirkung

Betroffenheit der nichtdominanten Schulter, fortgeschrittenes Lebensalter bei Erstluxation, Adipositas, Latarjet-Operationen

Vorliegen einer Osteoporose, fortgeschrittenes Lebensalter bei Erstluxation, Alkoholkonsum, arthroskopische Bankart-Operationen

Anzahl der Schulterluxationen, fortgeschrittenes Lebensalter bei Erstluxation, Alkoholkonsum, Sportarten mit Gewalteinwirkung

Insulinpflichtiger Diabetes mellitus, fortgeschrittenes Lebensalter bei Erstluxation, Drogenkonsum, J‑Span nach Resch

Sie planen bei einem 20-jährigen Drittligafußballspieler mit Profivertrag eine Schulterstabilisierung nach 3 Luxationen (5–10 % Knochenverlust am Glenoid). Sie beraten Spieler und Vereinsarzt bezüglich des Return to Sports (RTS) und der Rezidivinstabilität. Wie sind RTS-Parameter und Rezidivrate nach einer Latarjet-Operation (LAT) und einer arthroskopischem Bankart-Reparatur mit Kapsel-Shift (ABRK) in diesem Fall prognostisch einzuschätzen?

Nach einer LAT-Operation ist die RTS-Rate im Vergleich zur ABRK signifikant höher bei gleichem Rezidivrisiko.

Die RTS-Zeit ist nach einer ABRK geringer als nach einer LAT-Operation bei höherem Rezidivrisiko.

Die Rezidivrate ist nach einer LAT-Operation signifikant geringer und die RTS-Zeit signifikant kürzer.

Die RTS-Zeit beträgt 3 bis 5 Monate nach einer ABRK und 6 Monate nach einer LAT-Operation bei gleichem Rezidivrisiko.

Nach einer ABRK ist die Rezidivrate signifikant geringer und die RTS-Zeit signifikant kürzer.

Wie hoch wird der kritische Knochenverlust am Glenoid nach neuesten Erkenntnissen am ehesten definiert?

5–10 %

10–20 %

25–30 %

30–40 %

45–50 %

Welcher Nachteil ist bei einer arthroskopischen Bankart-Reparatur mit Kapsel-Shift + Remplissage (ABRK+R) beim Wurfsportler unbedingt in Erwägung zu ziehen?

Die Rezidivrate ist höher als bei anderen Verfahren.

Die RTS-Zeit ist länger als bei anderen Verfahren.

Die Außenrotationeinschränkung des Wurfarms.

Die RTS-Rate ist geringer als bei anderen Verfahren.

Die hohe intraoperative Komplikationsrate.
